# Incidentalome in Neurogenetics: Pathogenic Variant of *NSD1* in a Patient With Spinocerebellar Ataxia (SCA)

**DOI:** 10.3389/fgene.2018.00086

**Published:** 2018-03-14

**Authors:** Harvy Velasco, Diana Ramírez-Montaño

**Affiliations:** ^1^Medicina Faculty, Morphology Department, Universidad Nacional de Colombia, Bogotá, Colombia; ^2^Faculty of Health Sciences, Department of Basic Medical Sciences, Universidad Icesi, Cali, Colombia

**Keywords:** genetic incidentalome, diagnostics test, *NSD1*, whole-exome sequencing, late-onset sporadic ataxias

## Abstract

**Background:** Genetic studies of late-onset sporadic ataxias (>40 years of age) are not routinely indicated. For unresolved cases, next-generation sequencing (NGS) tools, such as whole-exome sequencing (WES), are available for a definitive diagnosis.

**Case presentation:** Our patient is a woman with a usual facial phenotype and anthropometry, who developed ataxia at 45 years of age, with no relevant family history and an initial clinical approach that ruled out common aetiologies. WES was performed when the patient was 54 years old. The results identified the heterozygous pathogenic variant c.248delA (p.N83MfsX4) in the nuclear receptor-binding SET domain protein 1 (*NSD1*; MIM 606681) gene (related to Sotos syndrome), which was not associated with ataxia and is not related to the patient's phenotype. Sanger sequencing of *NSD1* in two different laboratories confirmed the variant.

**Conclusions:** NGS findings generally offer valuable information that can be used for clinical decision-making. However, an incidental finding that leads to defining new clinical and bioethical actions is also possible. Consequently, the biological importance of this type of genetic “incidentalome” must be determined.

## Background

Whole-exome sequencing (WES) has changed the genetic analysis of monogenic diseases in the last decade. This diagnostic tool is used to assess both dysmorphological genetic syndromes and several neurological phenotypes, including Mendelian disorders associated with ataxia. WES has a diagnostic efficacy of >25%; (Baldridge et al., 2017), and it has been helpful to diagnose cases that did not have a definitive diagnosis. It has even facilitated the identification of new syndromes and genes associated with nosological events (Bamshad et al., 2011). In addition to determining a specific diagnosis, WES can also diagnose the coexistence of genetic conditions, which occur in 4.5–7% cases (Farwell et al., 2015), and it can incidentally identify clinically relevant genetic variants unrelated to the patient's phenotype (Green et al., 2017).

There has been a massive increase in incidental genetic findings in studies involving next-generation sequencing (NGS; WES or targeted sequencing) (Green et al., 2017). The clinical term “incidentalome” usually refers to incidental findings in images without clinical suspicion (Kohane et al., 2012). It is also used to describe findings that are not expected in genetic analysis, and genetic incidentalomes are currently estimated to occur in 5–9% cases (Kohane et al., 2012). However, reports of on such incidentalomes in the literature are few (Vu et al., 2016), and there have been no reports on the underlying neurological pathology.

In recent years, the neurogenetic aetiology of adults has progressed significantly because of the use of NGS technologies (Baldridge et al., 2017). For example, at least 60 types of hereditary ataxia are currently recognized, with an overall prevalence of 15–20 in 100,000 individuals; the most common forms show autosomal dominant (AD) inheritance (Mancuso et al., 2014).

In cases of suspected hereditary ataxias, the approach can be initiated by exploring clinical elements such as the age of onset, the inheritance mechanism, and the presence of “pure” ataxic symptoms or additional symptoms. Imaging findings should also be analyzed (Klockgether, 2010). Genetic studies may be indicated according to the patient's clinical signs and may include a triplet expansion analysis to study AD ataxia (Mancuso et al., 2014). However, despite these molecular tools, up to 50% adult patients with late-onset sporadic ataxias (>40 years of age) are not diagnosed and are classified as late-onset-idiopathic ataxia (Klockgether, 2010).

This paper reports the case of a 54-year-old patient with a history of late-onset idiopathic spinocerebellar ataxia (SCA) in which a definitive diagnosis was not established after biochemical, imaging and genetic studies. WES reported an “incidentalome” in nuclear receptor-binding SET domain protein 1 (*NSD1*; MIM #606681) that apparently does not explain the clinical status of the patient.

## Case presentation

Our case was a 54-year-old female patient from northern Colombia with a clinical history of 7 years of progressive gait disorder with a slow evolution, frequent falls, vertigo, and dysarthria but without tremor. She did not have a history of alcohol or other toxic substance consumption, trauma or cerebrovascular accidents, or a history of other symptoms or associated chronic pathologies. Family history was negative for parental consanguinity or similar neurological presentation. An ophthalmological examination ruled out retinal and oculomotor involvement.

**Figure 1:**
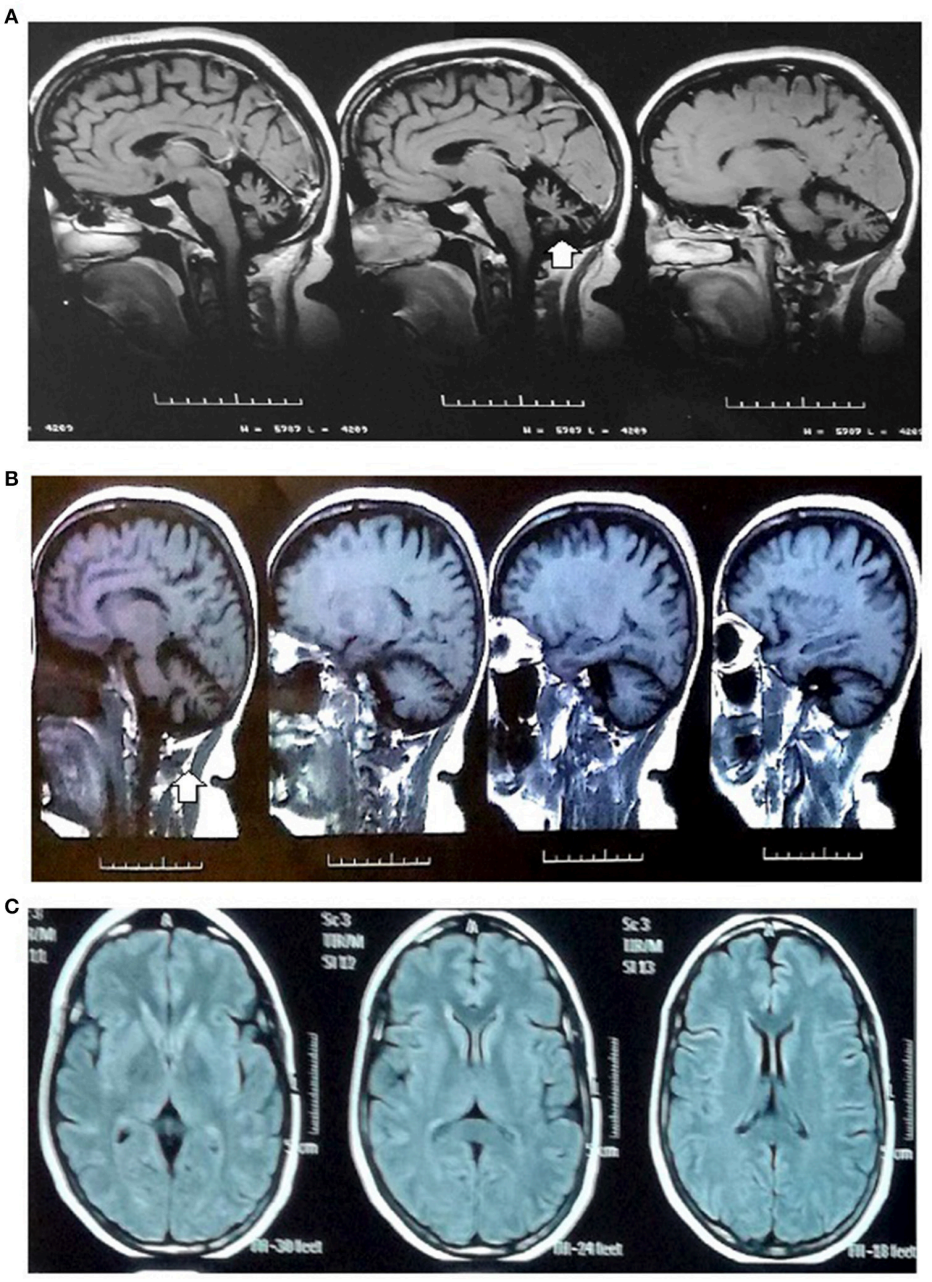
MRI images. MRI images (1.5 tesla): **(A,B)** Sagittal sections in the T1-weighted MRI images: global cerebellar atrophy with a marked enlargement of the subarachnoid space, thinning of cerebellar folds and vermis (arrow) related to diffuse cerebellar atrophy. **(C)** Axial section in the T1 sequence: cerebral white matter and ventricles without alterations.

The initial MRI at age 47 years showed evidence of cerebellar atrophy as the only relevant finding. The patient underwent several annual follow-up MRI examinations; the last MRI examination at age 53 years did not show any additional findings (Figure 1). Complementary neurological studies such as electromyography and nerve conduction velocities were performed at age 47 years.

At age 54 years, the patient had a height of 161 cm, a weight 67 kg, a body mass index of 24.7, and a head circumference of 56 cm; her facial appearance and phenotype were normal (Supplementary Figure 1). Her gait was slightly ataxic with a large walking base, greater involvement of toe-to-heel walking, and lateropulsion to the right side. A speech articulation disorder was also noted. Other findings include dysmetria and adiadochokinesia. There was no alteration of deep tendon reflexes or strength, the Romberg and Babinski signs were negative and there was no cranial nerve involvement, nystagmus, or sensory loss.

Complementary blood examination yielded the following results: normal vitamin B12 (309 pg/mL) and vitamin E (17 μg/mL) levels, normal TSH (3.48 mIU/L) levels, and negative HIV and VDRL tests. Other studies performed to rule out normal paraneoplastic syndrome (50 years), including chest X-ray, ultrasonography of the abdomen and mammography, showed no abnormalities.

As an initial genetic study, we performed triplet replication analysis by polymerase chain reaction fragment amplification (at age 49 years) to assess *Ataxin1* (SCA 1), *Ataxin2* (SCA 2), *MJD1* (SCA 3), and *CACNA1A* (SCA 6). The number of expansions were within the normal range for each gene studied. Karyotype or array-CGH studies were not indicated in the absence of clinical features (major and minor anomalies) that supported chromosomal abnormalities; furthermore, indel-type mutations have not been reported in ataxia-related genes. WES was also performed at age 53 years.

**Figure 2:**
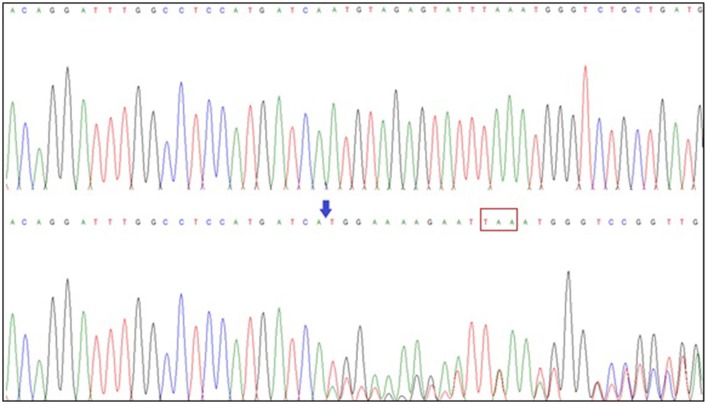
*NSD1* gene sequencing. Exon 2 sequence of the *NSD1* gene (superior: normal; inferior: patient sequence) showing the deletion of adenine (blue arrow) at position 247 (c.247delA), which has an effect on the protein and generates a premature stop codon at amino acid 87 (red box).

WES was performed on the sequencing coding and flanking intronic regions using the illumine HiSeq2500/4000 system. Illumina CASAVA was used to demultiplex the sequencing reads. The trimmed reads were mapped to the human reference genome (GRCh38). Variants were identified using Samtools and VarScan. Only variants (SNV/small indels) in the coding region and flanking intronic region (±8 bp) with a minor allele frequency (MAF) <1.5% were evaluated. Known disease-causing variants (according to HGMD) was evaluated in areas up to ±30 bp of the flanking regions and up to 5% MAF. MAFs were obtained from the following databases: 1000 genomes, dbSNP, Exome Variant Server, ExAC and an in-house database. The average collage comprised 100 × of at least 30 high-quality sequencing reads per base (~95% of the covered target bp >20×). This examination revealed a variant c.247delA (p.N83MfsX4) in the *NSD1* gene. No other variants were identified by this methodology. This finding was confirmed by Sanger sequencing *NSD1* gene (All exons). A likely pathogenic variant was found in the heterozygous state, c.247delA (p.N83MfsX4), which led to a premature stop codon at amino acid 87 (Figure 2). This result was reconfirmed by a European laboratory. Variant functional prediction software tools Mutation taster, Condel, SIFT, and FATHMM classified it as a Damaging variant (disease causing).

The Ethics Committee of the Universidad Nacional de Colombia (CE-010/2013) approved this study. The patient provided full written consent for publication.

## Discussion and conclusions

A molecular approach to ataxias is indicated when there is suspicion of a monogenic component, although it is not routinely indicated for late-onset sporadic ataxias (>40 years of age) (Klockgether, 2010). The usual form of presentation is the “pure” cerebellar form, similar to the case reported here. For this reason, we analyzed the group of SCAs, particularly SCA6 (Klockgether, 2010) and types 1, 2, and 3 because of their population frequency; the results showed a normal expansion size.

WES can identify causality in diseases with high clinical and genetic heterogeneity, such as ataxia. Its diagnostic performance is close to 40% (Farwell et al., 2015; Baldridge et al., 2017). In this case, the results did not show the causality; instead, they identified an incidentalome, which generated uncertainty regarding the genetic counseling and subsequent management of the pathology.

In 2014, the American College of Medical Genetics and Genomics (ACMG) (Green et al., 2017) defined 56 actionable genes with informational obligation for incidental findings; *NSD1* is not included in this list. The incidental finding of a pathogenic variant in *NSD1* by WES in our patient with ataxia raises the possibility of a false-positive finding because this gene is implicated in Sotos syndrome, which is a pathology with a recognizable phenotype (Douglas et al., 2003) not matching the phenotype of the presented case. In addition, there have been no reports of ataxia in patients with Sotos syndrome to date.

The *NSD1* gene is located at 5q35.3 and causes Sotos syndrome in 95% individuals. Frameshift mutations in this gene are always considered pathogenic (Douglas et al., 2003). The mutation reported here generated a premature stop codon at amino acid 87 that could severely affect the functionality of the protein. In a previous study in which the molecular pathways that are generated from *NSD1* interactions in STRING were analyzed, correlation or overlap with pathways involved in the pathogenesis of ataxia was not shown; (Nibbeling et al., 2017) the study showed that the pathways are limited to functions related to possible epigenetic regulatory events.

Kohane et al. (2012) established four elements that must be analyzed to differentiate an incidental finding from a false-positive one: erroneous annotation, sequencing error, incorrect estimation of penetrance, and multiple hypotheses when conducting the test. With regard to the first two elements, the variant found by WES was independently confirmed by two different laboratories through Sanger sequencing, and both laboratories reported the same results. Regarding the incorrect estimation of penetrance, Sotos syndrome displays full penetrance (100%) and highly variable expressivity (Douglas et al., 2003). Furthermore, the generational analysis of the patient ruled out the existence of attenuated phenotypes of related syndromes.

In conclusion, the relevance and clinical impact of incidental findings depend on several factors such as the exact definition of the patient's phenotype, the validation and relevance of molecular tests, and the appropriate allocation and classification of the variants (Green et al., 2017). In asymptomatic patients, there is no strong evidence to guide clinical behavior (Kohane et al., 2012). This incidental finding creates an interesting scenario with two possibilities: a new molecular mechanism that allows associating this gene with ataxia or a scenario of incomplete penetrance not previously reported in Sotos syndrome.

## Author contributions

All authors listed have made a substantial, direct and intellectual contribution to the work, and approved it for publication.

### Conflict of interest statement

The authors declare that the research was conducted in the absence of any commercial or financial relationships that could be construed as a potential conflict of interest.

## Supplementary material

Supplementary Figure 1 Facial phenotype. Phenotype no compatible with Sotos syndrome: no macrocrania or overgrowth.

## Abbreviations

ACMG: American College of Medical Genetics and Genomics
AD: autosomal dominant
Ngs: next-generation sequencing
SCA: spinocerebellar ataxia
WES: whole-exome sequencing
HGMD: the human gene mutation database.
