# Supplementary figures and images for: Incidentalome in Neurogenetics: Pathogenic Variant of NSD1 in a Patient With Spinocerebellar Ataxia (SCA)
Source: Front Genet. 2018 Mar 14;9:86. doi: 10.3389/fgene.2018.00086 (PMC5861145; doi:10.3389/fgene.2018.00086)

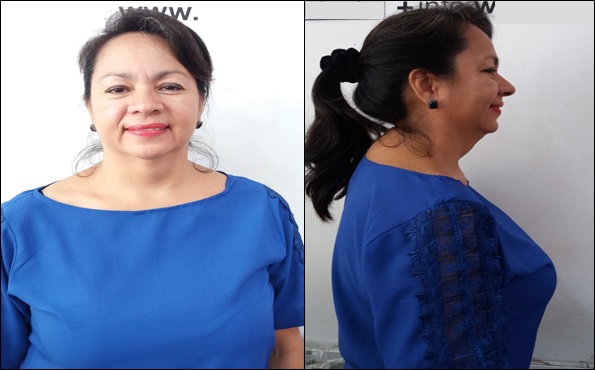

Supplement: Supplementary Figure 1 — Facial phenotype. Phenotype no compatible with Sotos syndrome: no macrocrania or overgrowth. [file Image1.JPEG]
